# Causal association between body mass index and autoimmune thyroiditis: evidence from Mendelian randomization

**DOI:** 10.1186/s40001-023-01480-1

**Published:** 2023-11-16

**Authors:** Jinlong Huo, Yaxuan Xu, Jie Yu, Youming Guo, Xiaochi Hu, Dong Ou, Rui Qu, Lijin Zhao

**Affiliations:** 1 Department of Breast and Thyroid Surgery, The Third Affiliated Hospital of Zunyi Medical University (The First People’s Hospital of Zunyi), Zunyi, Guizhou China; 2 Department of General Surgery, Digestive Disease Hospital, Affiliated Hospital of Zunyi Medical University, 149 Dalian Road, Huichuan District, Zunyi, Guizhou China; 3 Department of Oncology, The First People’s Hospital of Zunyi), The Third Affiliated Hospital of Zunyi Medical University, Zunyi, Guizhou China

**Keywords:** Mendelian randomization, Autoimmune thyroiditis, Body mass index, Causal association, Genetic variants

## Abstract

**Background:**

Recent studies have reported associations between body mass index (BMI) and various autoimmune disorders. However, it is still uncertain whether there exists a direct cause-and-effect relationship between BMI and autoimmune thyroiditis (AIT). The aim of our study is to investigate the causal association between BMI and AIT.

**Methods:**

We conducted a two-sample summary data Mendelian randomization (MR) analysis using genome-wide association studies (GWAS) summary statistics data related to BMI as exposure, and GWAS summary statistic data sets for AIT as the outcome. Robustly associated single-nucleotide polymorphisms (SNPs) for BMI were selected as instrumental variables (IVs). We used the inverse variance weighted (IVW) method as the primary method and performed other MR methods such as MR-Egger regression, weighted median, simple mode, and weighted mode analyses for further validation. The slope of MR-Egger regression was used to correct for pleiotropy and provide estimates of causality. The *p*-value for the intercept in MR-Egger was utilized to detect any directional pleiotropic effects. Heterogeneity and sensitivity analyses were performed to assess the robustness of our findings.

**Results:**

Seventy-eight SNPs were selected from GWAS on BMI as the IVs. Our MR analysis using the IVW method showed a potential causal association between BMI and AIT (OR = 3.071, 95% CI 1.324–7.118). Findings from other MR methods are non-significant, although the direction of effect is consistent. There was no evidence that the result was affected by genetic pleiotropy (MR-Egger regression intercept = 0.01, SE = 0.00025, *p* = 0.719). Heterogeneity and sensitivity analyses revealed no significant heterogeneity among SNPs, and no single SNP drove the observed associations.

**Conclusion:**

Our findings suggest a potential causal association between BMI and AIT, which may provide a basis for further investigation into the relationship between BMI and AIT. Further studies are required as only the IVW method shows significant results, and the case sample size is small.

**Supplementary Information:**

The online version contains supplementary material available at 10.1186/s40001-023-01480-1.

## Introduction

Autoimmune thyroiditis (AIT), particularly Hashimoto's disease, is the most common autoimmune thyroid disease and is estimated to affect about 1–5% of the general population [1]. As the most common autoimmune inflammatory disease of the thyroid gland, AIT is also the main cause of autoimmune hypothyroidism [2]. Importantly, AIT-induced hypothyroidism typically persists permanently and usually necessitates lifelong thyroid hormone replacement therapy [2, 3]. In addition, as thyroid tissue is gradually destroyed and production of thyroid hormones decreases, it can cause various symptoms such as fatigue, weight gain, constipation, feeling cold, dry skin, depression, muscle pain, and reduced tolerance to exercise [4, 5].

The treatment for AIT depends on the severity of the condition and the symptoms experienced by the patient. Clinical evidence has shown that myo-inositol and selenium can be effective in treating AIT [6–8]. A systematic review and meta-analysis have found that vitamin D supplementation may have a beneficial effect on thyroid autoantibody levels in the treatment of AIT [9]. In addition, Glycyrrhizin, a direct HMGB1 antagonist, has been shown to ameliorate inflammatory infiltration in a murine model of AIT via inhibition of TLR2-HMGB1 signaling [10]. These factors may influence the development of AIT. Although research has implicated oxidative stress and psychiatric disorders as potential risk factors for developing AIT [11, 12], the exact mechanisms underlying this relationship are not well understood.

Adipocytes in adipose tissue produce adipokines, which can have either pro-inflammatory or anti-inflammatory effects in various tissues and organs, including the thyroid gland [13–15]. Adipocytes can also recruit and activate immune cells, promoting the release of pro-inflammatory mediators, and contributing to the development of chronic inflammatory conditions [16, 17]. Recent studies have reported that adipocytes possess the ability to engage in direct interactions with immune cells, either through cell-to-cell contact or via the release of extracellular vesicles containing microRNAs and other molecules. These interactions have been found to modulate the activity of immune cells and subsequently impact the magnitude of the immune response [18–21].

Body mass index (BMI) is a commonly used measure of body fatness and is calculated by dividing an individual's weight in kilograms by the square of their height in meters. It serves as a proxy for overall adiposity and is widely used in epidemiological studies to assess the relationship between obesity and various health outcomes [22, 23]. Consequently, it is hypothesized, based on the aforementioned research, that individuals with elevated BMI may be at an increased susceptibility for the development of AIT. This supposition finds support in observational studies [24, 25]. However, these types of studies can be influenced by biases such as reverse causation and residual confounding, which may limit our understanding of the impact of BMI on AIT.

Mendelian randomization (MR) is a statistical technique that uses genetic variants as instrumental variables (IVs) to evaluate the causal relationship between an exposure factor and an outcome [26]. This method can provide stronger evidence of causality than observational studies since it can minimize confounding and reverse causation bias [26]. Several MR studies have demonstrated the causal relationship between BMI(as the exposure) and numerous immune-related diseases [27, 28]. However, to our knowledge, no relevant studies have reported the causal effect of BMI on the risk of AIT.

In this study, we performed a two-sample summary data MR analysis to comprehensively investigate the causal relationship between BMI (as the exposure) and AIT (as the outcome).

## Materials and methods

### Study design

Using past explorations about BMI (Additional file 1: Table S1) as a reference, we combined and rectified these genetic variants (i.e., single-nucleotide polymorphisms (SNPs)) data for use as instrumental variables alongside AIT-related genome-wide association study (GWAS) summary information available (Additional file 2: Table S2). We then conducted a two-sample MR study to determine the relationship between BMI and AIT. We carried out our statistical analysis by utilizing the two-sample MR package (version 0.5.4) from the R program (version 4.3.0). We only utilized summarized-level data in our study, which meant ethical approval was unnecessary.

### Data sources and selection of genetic variants

GWAS summary statistics data related to BMI were obtained from the IEU OpenGWAS project (https://gwas.mrcieu.ac.uk) as exposure, which includes 339,224 individuals of European ancestry with GWAS ID number ieu-a-2 [29]. Two-sample MR study was conducted using genetic variants (SNPs) robustly associated with BMI at *p* < 5 × 10^−8^. To obtain independent SNPs with a more relaxed threshold, SNPs were clumped and discarded based on linkage disequilibrium (LD) *r*^2^ > 0.001 using reference data from the 1000 Genomes Project for European ancestry. F-statistics were calculated to quantify the strength of genetic variants, and SNPs with a value less than 10 were discarded for their insufficient strength [30]. The selected SNPs were used as instrumental variables (IV) in the current MR study.

The publicly available summary statistic data sets of a GWAS for AIT were selected as the outcome (https://gwas.mrcieu.ac.uk/datasets/finn-b-E4_THYROIDITAUTOIM/), which included 187,928 individuals (cases = 244, controls = 187,684) of European ancestry with GWAS ID number finn-b-E4_THYROIDITAUTOIM. Details of the IVs in the outcome were extracted and merged with exposure data. To evaluate whether the IVs were associated with AIT risk factors, PhenoScanner (http://phenoscanner.medschl.cam.ac.uk/phenoscanner) was used, which provides SNP phenotype information [31, 32]. Thus, we ensure that we guarantee the principles of relevance, independence and exclusion restriction required by MR [33]. For missing data in our study, we will delete missing data if the proportion of missing data is small, However, if the proportion of missing data is large, this method may reduce the sample size and affect the reliability of the results.

### Statistical analysis for Mendelian randomization

All statistical analyses were conducted using the Two Sample MR package in R software (version 4.3.0). The inverse variance weighted (IVW) method was utilized as the primary statistical method as it provides the most accurate estimate [34]; To enhance the accuracy of our assessment, we conducted additional MR methods including MR-Egger regression, weighted median, Simple mode, and Weighted mode analyses to further validate our findings [35–37]. Additionally, we used the slope of MR-Egger regression to correct for pleiotropy and provide estimates of causality [36]. The *p*-value for the intercept in MR-Egger was utilized to detect any directional pleiotropic effects [35].

### Heterogeneity and sensitivity analysis

We employed the IVW and MR-Egger methods to detect heterogeneity for each SNP. The degree of heterogeneity between SNPs was evaluated through the heterogeneity statistic Q and Funnel plot [38]. Furthermore, a leave-one-out sensitivity analysis was performed to determine if the results of the MR investigation were influenced by a single SNP [38].

## Results

### Instrumental variables for Mendelian randomization

**Fig. 1:**
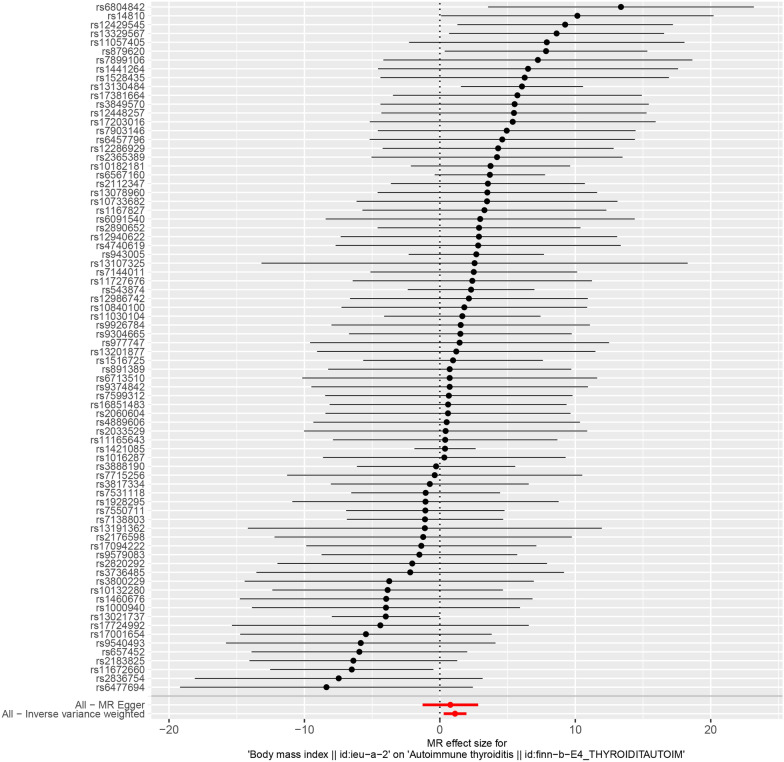
Forest plot of single SNPs associated with BMI on AIT. *BMI* body mass index, *AIT* autoimmune thyroiditis

This study utilized 78 independent SNPs from GWASs on BMI as Ivs (without missing data). These SNPs were positively related to AIT, which is illustrated in Fig. 1 and Additional file 2: Table S2. Each SNP had an F statistic exceeding 10, indicating a decreased possibility of weak instrument bias (Additional file 2: Table S2). Furthermore, querying the PhenoScanner curated database did not reveal any noteworthy connections between individual SNPs and AIT.

### Mendelian randomization results

**Fig. 2:**
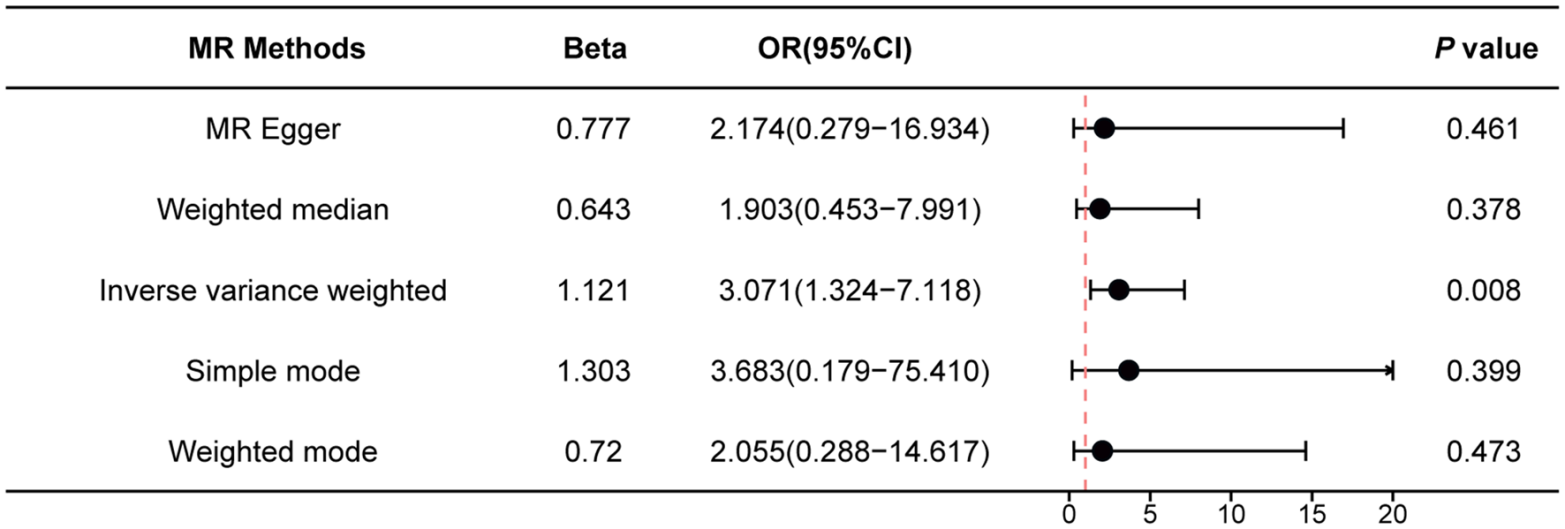
Mendelian randomization estimates from each method of assessing the causal effect of BMI on the risk of AIT. *BMI* body mass index, *AIT* autoimmune thyroiditis, *Beta* beta coefficient, *OR* odds ratio

**Fig. 3:**
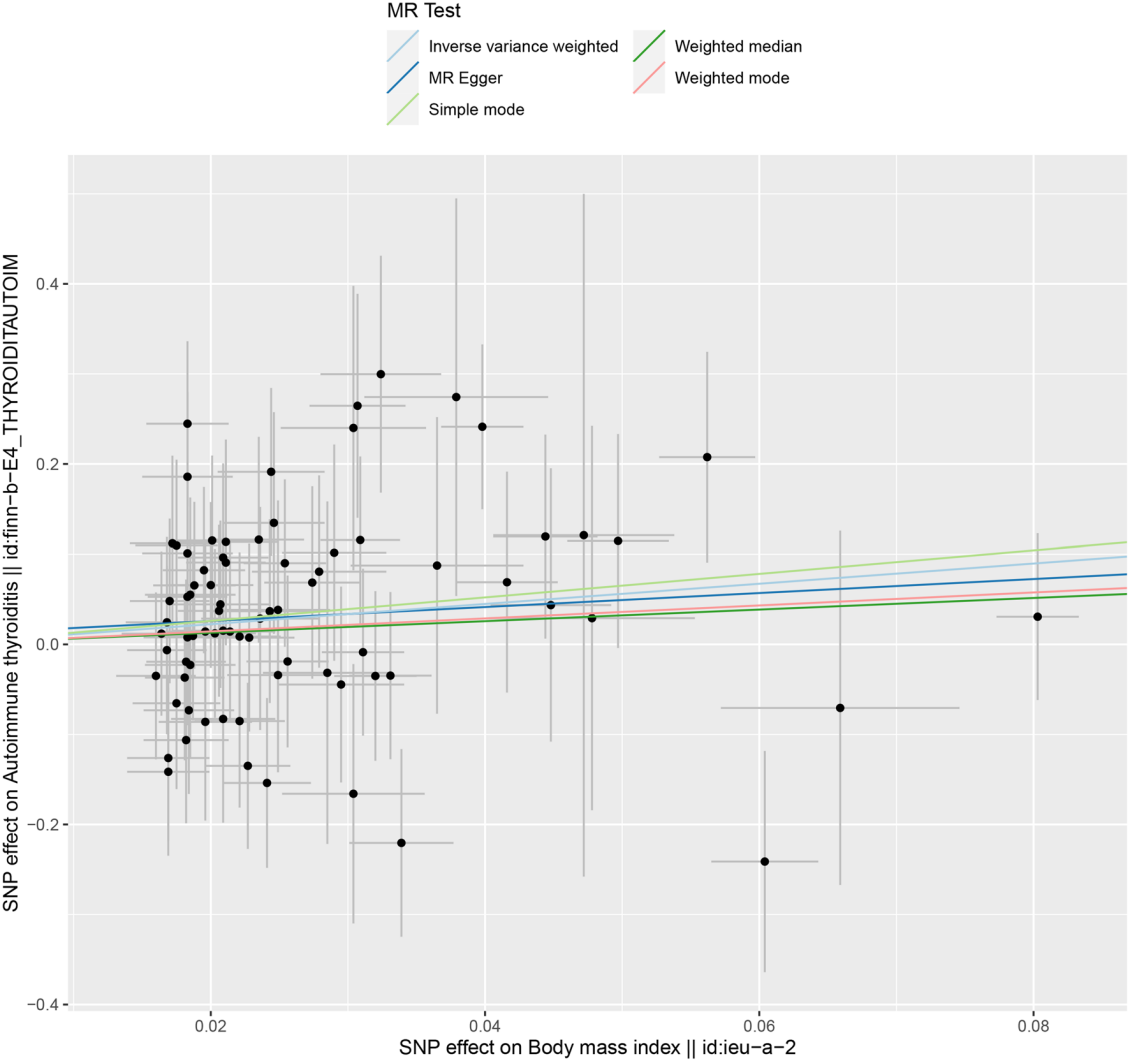
Scatter plot of SNPs associated with BMI and the risk of AIT. The plot presents the effect sizes of the SNP-BMI association (x-axis, SD units) and the SNP-AIT association (y-axis, log (OR)) with 95% confidence intervals. The regression slopes of the lines correspond to causal estimates using the five Mendelian randomization (MR) methods (the IVW method, weighted median estimator, simple mode, weighted mode and MR-Egger). *BMI* body mass index, *AIT* autoimmune thyroiditis

The IVW method showed evidence to support a causal association between BMI and AIT (OR = 3.071, 95% CI 1.324–7.118), and other MR methods showed the tendency of a causal association between BMI and AIT but without statistical significance (Figs. 2 and 3). The results of the MR analysis may support a potential causal association between BMI and AIT. The intercept represents the average pleiotropic effect across the genetic variants (the average direct effect of a variant with the outcome). An intercept that differs from zero (the MR‐Egger test) is indicative of directional pleiotropy. There was no evidence that the result was affected by genetic pleiotropy (MR-Egger regression intercept = 0.01, SE = 0.00025, *P* = 0.719).

### Heterogeneity and sensitivity test

**Fig. 4:**
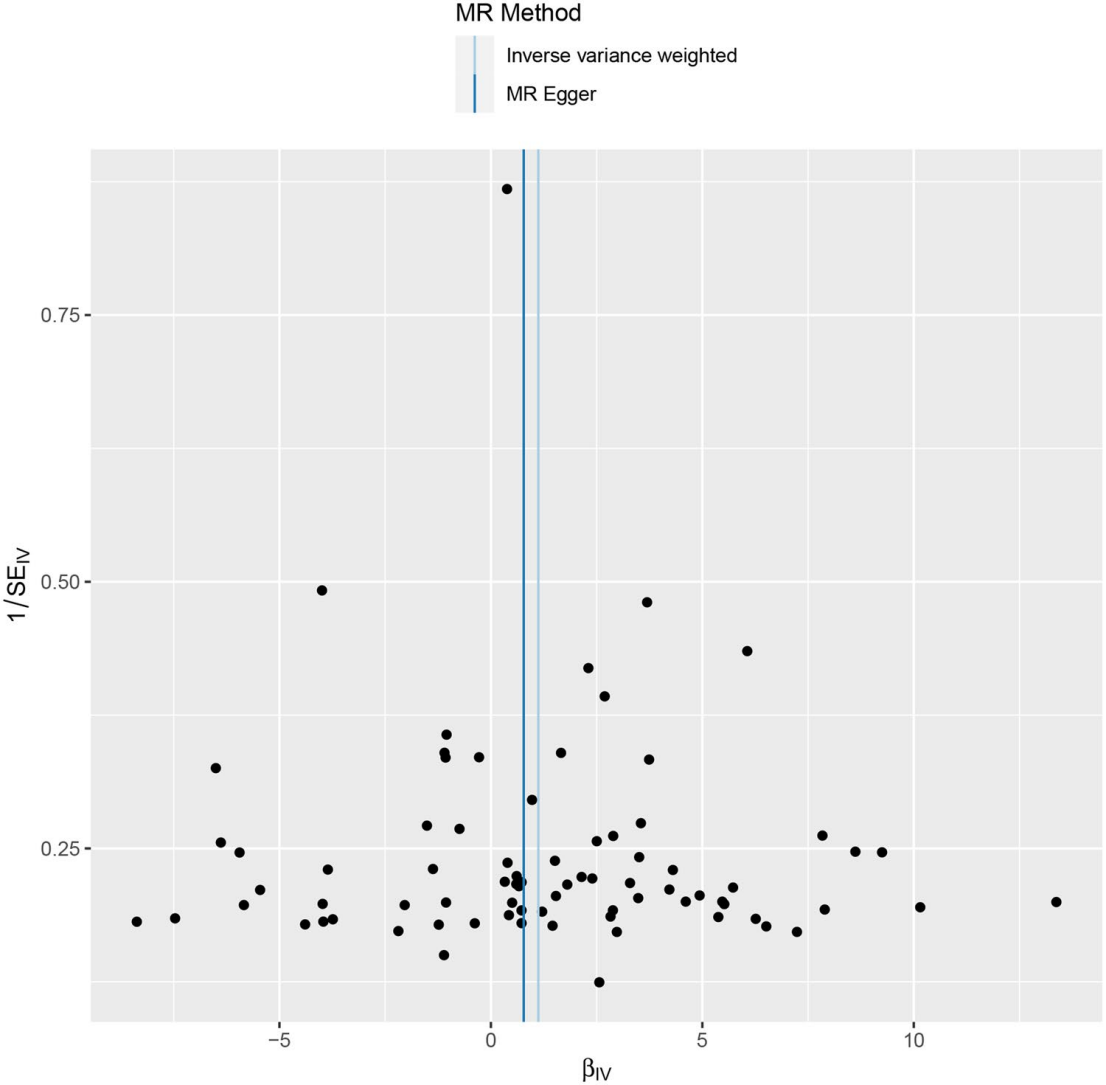
The funnel plot of Mendelian randomization analysis. The funnel plot appears symmetrical, indicating no evidence of heterogeneity

**Fig. 5:**
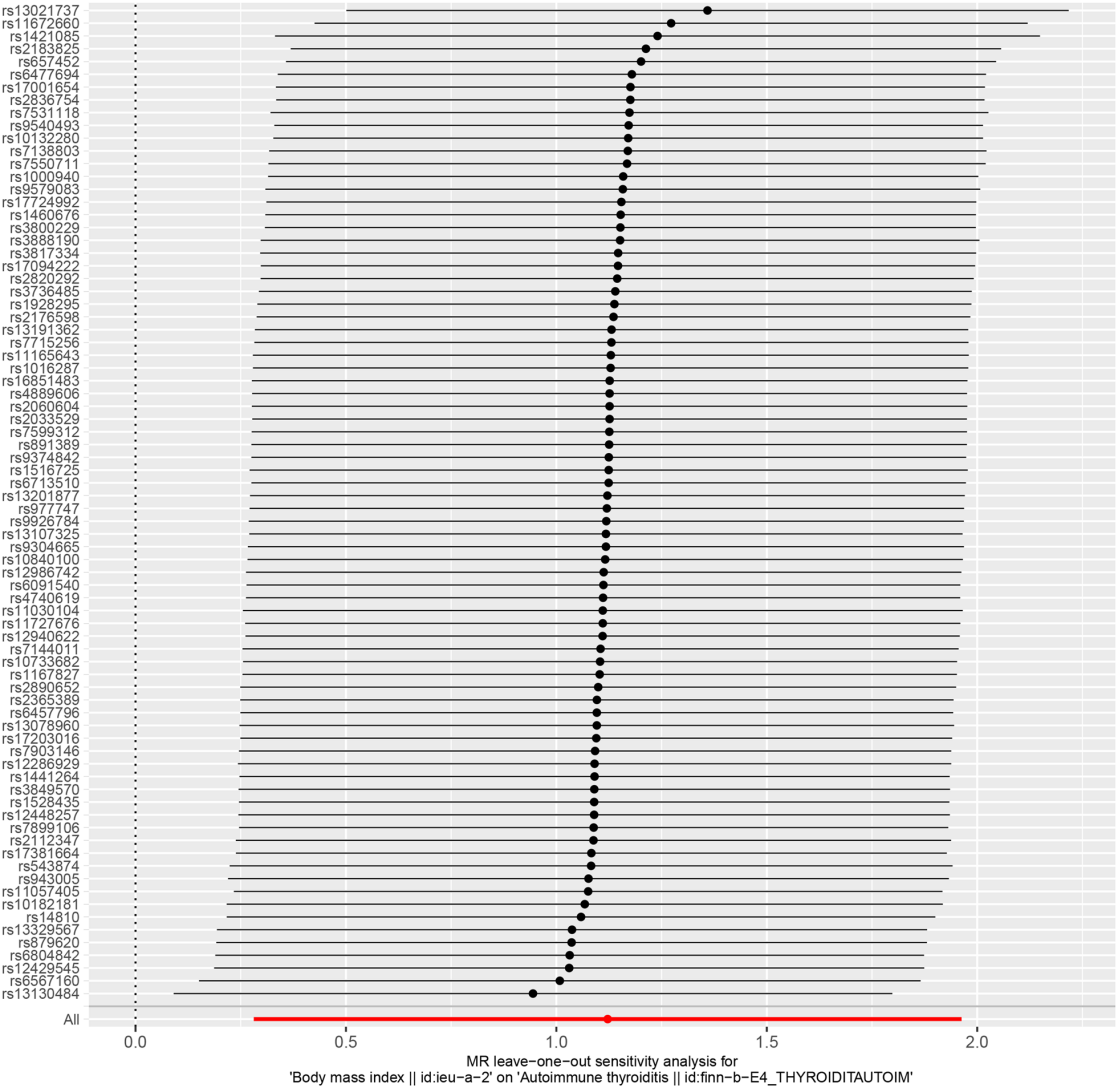
Leave-one-out of SNPs associated with BMI and their risk of AIT. Each black point represents the result of the IVW MR method applied to estimate the causal effect of BMI on AIT. *BMI* body mass index, *AIT* autoimmune thyroiditis, *IVW* inverse variance weighted, *MR* Mendelian randomization

The Q test, which incorporated both the IVW and MR-Egger methods, revealed no significant heterogeneity among SNPs (Table 1), Additionally, the funnel plot showed a basically symmetrical shape(Fig. 4), suggesting that the results are robust. The leave-one-out method demonstrated that no single SNP disproportionately influenced the observed associations (Fig. 5).

**Table 1:** Heterogeneity test among SNPs

Method	Q	df	*p*-value
Inverse variance weighted	77.35	77	0.570
MR-Egger	77.22	76	0.593

## Discussion

This study utilized the MR approach to assess the relationship between BMI and AIT. For our primary analysis, we employed the IVW method, which has been shown to have high sensitivity in detecting causal effects. Our IVW results provide evidence supporting a potential causal association between BMI and AIT. We also conducted additional analyses, including the weighted median, MR-Egger, and weighted mode methods, in the two-sample MR framework. These analyses revealed a trend towards causality between BMI and AIT, although statistical significance was not observed. Nonetheless, the consistency of our results across various methods strengthens the robustness of our findings.

Previous studies have suggested a potential link between BMI and AIT, where higher BMI levels have been associated with an increased risk of developing AIT [24, 25]. However, these studies were observational, meaning that they examined the relationship between BMI and AIT in real-world settings without interventions or control groups. Note that observational studies have some limitations, such as the presence of confounding factors and the possibility of reverse causation. Confounding factors are variables that can independently influence both BMI and AIT, which makes it challenging to determine if BMI directly causes AIT. On the other hand, reverse causation suggests that AIT could actually lead to weight gain and higher BMI, rather than higher BMI causing AIT. While these observational studies offer valuable insights, they cannot definitively establish a causal link between BMI and AIT. By utilizing the Mendelian randomization approach, we were able to overcome these limitations and provide more robust evidence supporting a causal link between BMI and AIT.

**Fig. 6:**
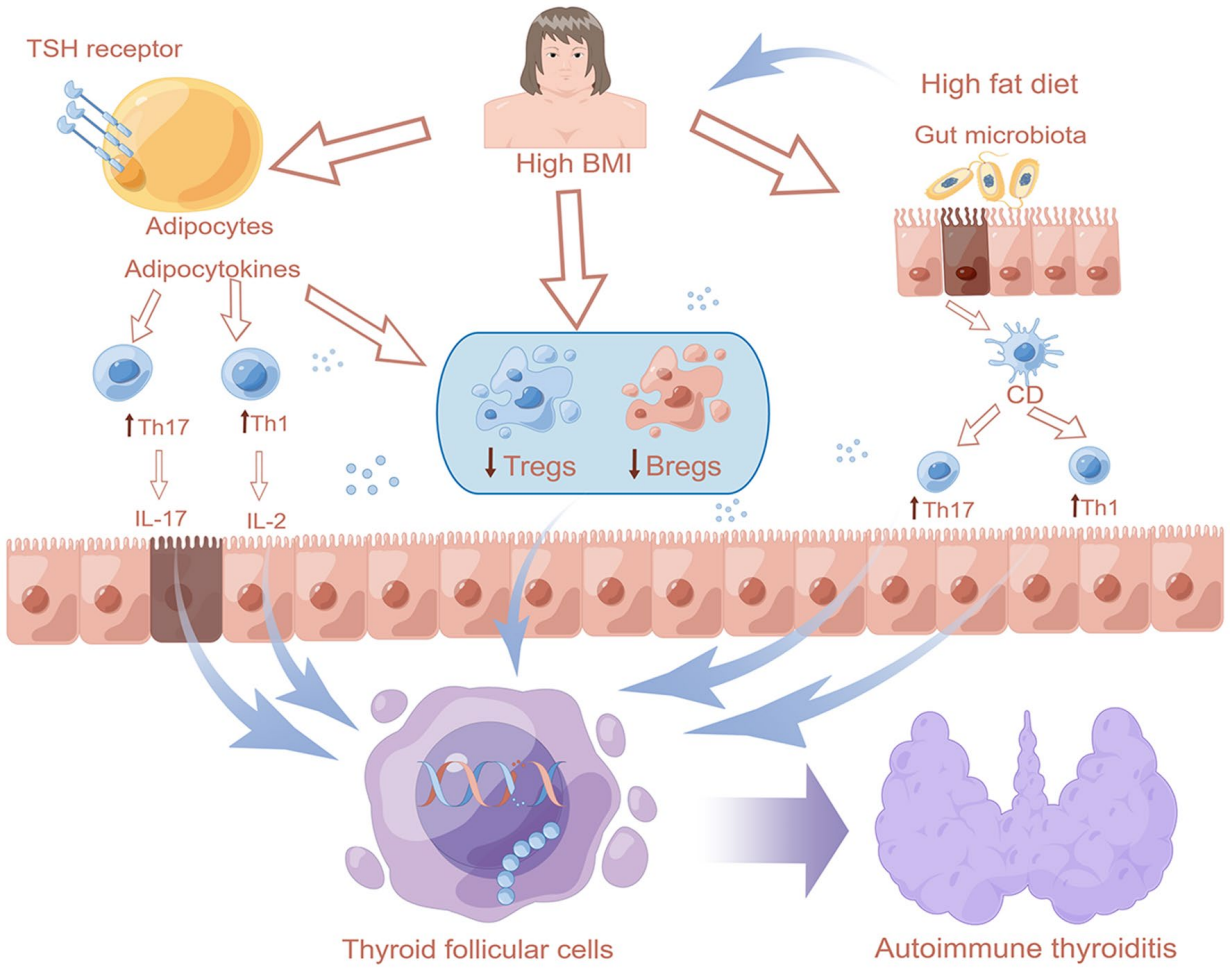
Summary of several mechanisms by which BMI affects the pathogenesis of AIT reported in the literature. *Bregs* regulatory B cells, *Tregs* regulatory T cells, *Th17* T-helper 17 cells, *Th1* T-helper 1 cells, *BMI* body mass index, *AIT* autoimmune thyroiditis

The mechanisms linking BMI and AIT are unclear. Research suggests that obesity can lead to gut dysbiosis, an alteration in the gut microbiota composition, which can trigger autoimmune diseases such as AIT [38, 39]. Dysbiosis can activate immune cells and produce pro-inflammatory cytokines as the gut microbiota plays a crucial role in regulating the immune system [39, 40]. Obesity can also hinder human self-tolerance mechanisms by promoting pro-inflammatory processes and reducing the number of Bregs and Tregs, leading to increased Th17 and Th1 cells that create an environment favorable for autoimmune disorders [14]. In Hashimoto's thyroiditis, adipose tissue enlargement in patients with high BMI results in the release of monocyte chemoattractant protein (MCP) from podocytes and monocytes that play a feedback role based on thyroid hormones [24]. Adipokines, which are biologically active substances derived from adipocytes, are involved in regulating metabolism, hormonal balance, and immune response [41, 42]. Obesity exacerbates the severity of certain autoimmune diseases and reduces treatment response. Moreover, excess body fat may promote the progression of autoimmune diseases [24]. Therefore, we hypothesize that AIT in high BMI patients might develop via similar mechanisms. Obesity could contribute to AIT development, which in turn may result in autoimmune disease progression. The thyroid and adipose tissues may interact bilaterally since adipocytes express functioning thyroid-stimulating hormone (TSH) receptors, and TSH binding to its receptor on brown adipocytes can stimulate thermogenesis. A schematic diagram to illustrate this concept is shown in Fig. 6.

Our study identified multiple SNPs associated with both BMI and immune function and inflammatory signaling, suggesting that immune function and signaling pathways may mediate the relationship between BMI and AIT. These results support our proposed hypothesis. However, the question of causality between BMI and AIT remains unresolved since not all patients with AIT are obese.

Previous studies have indicated that obesity is positively correlated with AIT, with obese individuals having a higher prevalence of AIT compared to non-obese individuals. The mechanism behind this correlation is not fully understood, but it is hypothesized that increased leptin production in obese individuals may promote Th17 cell differentiation, thus contributing to the development of AIT. Using MR, our findings provided evidence supporting a causal relationship between BMI and AIT. This provides stronger support than the mere correlation observed in observational studies and meta-analyses [43–45]. Our results are consistent with these findings, indicating that a high BMI may increase the risk of AIT. However, MR studies have limitations since they assume that instrumental variables are only related to the outcome through the risk factor being studied and require robust genetic instrumentation and large sample sizes [46].

In this study, we conducted heterogeneity and sensitivity analyses to assess the reliability and validity of our findings. Firstly, we assessed the level of heterogeneity among SNPs by employing the Q-statistic based on the IVW and MR-Egger methods. Our results indicated that there was no significant heterogeneity among SNPs, which strengthens the reliability of our findings. This was also confirmed by funnel plots, which did not show trends towards bias. Secondly, we performed a leave-one-out sensitivity analysis to explore if any single SNP was responsible for influencing the MR findings. Our results showed that the links were not reliant on a particular SNP. Thirdly, we employed the MR-Egger regression approach to detect directional pleiotropy, which can negatively impact MR techniques. We used the intercept of MR-Egger regression to provide free-of-bias estimates of causality. The results indicated that there was no evidence of genetic pleiotropy affecting our outcomes.

However, it should be acknowledged that our MR analysis may have certain limitations. Firstly, the accuracy of genetic instruments is essential for the validity of the MR approach. Despite using a significant number of independent SNPs as IVs, there remains the possibility of weak instrument bias. Secondly, one limitation of this study is the relatively small number of cases of AIT in the GWAS summary dataset. This may limit the statistical power to detect significant associations in some of the analyses. Additionally, it is worth noting that only the IVW method showed significant results, while other MR methods did not provide consistent findings. Therefore, caution should be exercised when interpreting the results as the evidence is not entirely convincing across all MR methods used in this study. Lastly, population stratification may influence the findings of MR analysis, potentially causing spurious associations. Although the MR approach minimizes the risk of confounding, it cannot fully eliminate the possibility of residual confounding, which might impact the results. Therefore, the interpretation of the results of this study should be cautious, and further research is necessary to confirm the causal relationship between BMI and AIT.

Our study underscores the significance of sample size selection in GWAS and how utilizing the European population for both exposure and outcome groups can address some of the limitations in MR. By using the European population, we mitigated the impact of genetic heterogeneity and population structure on the validity of our study. Our analysis involved a substantial sample size, using GWAS summary statistics data from 339,224 individuals of European ancestry as the exposure factor and 187,928 individuals of European descent from the autoimmune thyroiditis GWAS summary statistics as the outcome factor, which significantly increased the study's power.

The results of this study have clinical significance in terms of identifying a possible causal link between BMI and AIT. If confirmed through further investigation, healthcare providers may need to consider BMI as a risk factor for AIT and screen patients with high BMI for AIT. From a public policy perspective, our study may provide additional evidence for promoting healthy weight management practices to prevent the development of AIT. However, it should be noted that the effect size of possible interventions cannot be determined from this study alone, highlighting the need for further research to investigate the impact of interventions such as weight loss on the risk of developing AIT.

In summary, our MR analysis suggests that there may be a causal link between BMI and an increased risk of AIT, indicating that obesity could contribute to the onset of AIT. Our results provide a basis for future research into the association between BMI and AIT.

### Supplementary Information

**Additional file 1.** SNPs are significantly associated with BMI (*p* < 5E-08).

**Additional file 2.** Data after BMI and AIT synergy.

## Data Availability

Not applicable.

## Abbreviations

BMI: Body mass index
AIT: Autoimmune thyroiditis
MR: Mendelian randomization
GWAS: Genome-wide association study
SNP: Single-nucleotide polymorphism
IV: Instrumental variable
OR: Odds ratio
SE: Standard error
TLR2: Toll-like receptor 2
HMGB1: High mobility group box 1
MCP: Monocyte chemoattractant protein
Bregs: Regulatory B cells
Tregs: Regulatory T cells
Th17: T-helper 17
Th1: T-helper 1
